# Oxidative Stress in Cardiovascular Diseases: Involvement of Nrf2 Antioxidant Redox Signaling in Macrophage Foam Cells Formation

**DOI:** 10.3390/ijms18112336

**Published:** 2017-11-05

**Authors:** Bee Kee Ooi, Bey Hing Goh, Wei Hsum Yap

**Affiliations:** 1 School of Biosciences, Taylor’s University, Subang Jaya, Selangor Darul Ehsan 47500, Malaysia; ooibeekee@gmail.com; 2 School of Pharmacy, Monash University Malaysia, Bandar Sunway, Selangor Darul Ehsan 47500, Malaysia

**Keywords:** cardiovascular diseases (CVD), atherosclerosis, oxidative stress, macrophages foam cells, nuclear factor erythroid 2-related factor 2 (Nrf2), scavenger receptor class B (CD36), scavenger receptor class A (SR-A), lectin-type oxidized LDL receptor 1 (LOX-1), ATP-binding cassette transporter A1 (ABCA1), ATP-binding cassette transporter G1 (ABCG1)

## Abstract

Oxidative stress is an important risk factor contributing to the pathogenesis of cardiovascular diseases. Oxidative stress that results from excessive reactive oxygen species (ROS) production accounts for impaired endothelial function, a process which promotes atherosclerotic lesion or fatty streaks formation (foam cells). Nuclear factor erythroid 2-related factor 2 (Nrf2) is a transcription factor involved in cellular redox homeostasis. Upon exposure to oxidative stress, Nrf2 is dissociated from its inhibitor Keap-1 and translocated into the nucleus, where it results in the transcriptional activation of cell defense genes. Nrf2 has been demonstrated to be involved in the protection against foam cells formation by regulating the expression of antioxidant proteins (HO-1, Prxs, and GPx1), ATP-binding cassette (ABC) efflux transporters (ABCA1 and ABCG1) and scavenger receptors (scavenger receptor class B (CD36), scavenger receptor class A (SR-A) and lectin-type oxidized LDL receptor (LOX-1)). However, Nrf2 has also been reported to exhibit pro-atherogenic effects. A better understanding on the mechanism of Nrf2 in oxidative stress-induced cardiac injury, as well as the regulation of cholesterol uptake and efflux, are required before it can serve as a novel therapeutic target for cardiovascular diseases prevention and treatment.

## 1. Introduction

Cardiovascular diseases (CVD) including coronary heart disease (CHD), myocardial infarction (MI), and stroke are the leading causes of death globally, accounting for 31% of all global deaths (17.7 million) in 2015 [1]. Atherosclerosis, a slow progressing chronic inflammatory disease characterized by accumulation of lipids in the arterial intima and infiltration of immune cells, is one of the leading causes of CVD [2,3]. Oxidative stress and inflammation are closely associated with CVD and acute coronary syndromes [4,5]. Immune cells such as macrophages and dendritic cells are most often found in the intimal atherosclerotic lesions where they contribute to the inflammatory microenvironment of the lesions. Recruitment and retention of immune cells in atherosclerotic plaque leads to the production of cytokines, as well as other pro- and anti-inflammatory mediators that regulate atherosclerosis and chronic inflammation that accompanies this process [6]. Inflammation contributes to coronary disease by inducing the initiation and progression of atherosclerotic plaque, plaque rupture, and thrombosis (atherothrombosis). In addition, inflammation may also occur as a consequence of oxidative stress due to increased reactive oxygen species (ROS) and reactive nitrogen species (RNS) [4,5]. Oxidation of lipoproteins induced by ROS can amplify oxidized low density lipoproteins (oxLDL) formation and uptake by macrophages. Accumulation of oxLDL creates a foamy appearance in macrophages (foam cells). Studies have shown that increased levels of oxLDL-positive macrophages or foam cells formation relate to plaque instability in human coronary atherosclerotic lesions [7,8].

**Figure 1:**
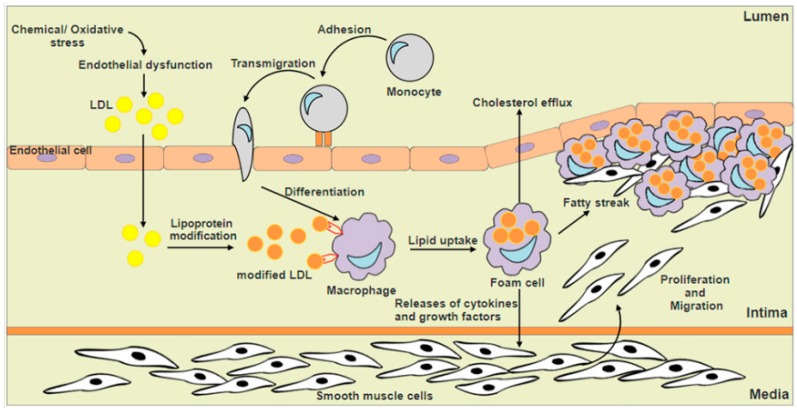
Macrophage foam cells formation and fatty streak development. Increased reactive oxygen species (ROS) production and oxidative stress induce endothelial dysfunction, which increases the permeability of endothelium and allows for the entry of low density lipoproteins (LDL) into the arterial intima layer. LDL within the intima layer may undergo oxidative modification, which results in endothelial cell activation, leading to the expression of chemoattractant factors and cytokines that facilitate the recruitment of monocytes from lumen into the arterial intima. Upon entering the arterial intima, monocytes are differentiated into macrophages which may internalize modified LDL, creating a foamy appearance within the macrophages, also known as foam cells. Macrophage foam cells produce a variety of cytokines and growth factors that stimulates the infiltration and proliferation of smooth muscle cells from the media to the arterial intima, which results in the thickening of the arterial walls where they transform the fatty streak into a stable plaque.

Macrophages contribute to plaque development by lipid retention that converts them into foam cells (Figure 1). Foam cells accumulate to create fatty streaks and contribute to the architecture of advanced plaques. Macrophage foam cells produce a variety of cytokines and growth factors such as interleukin-1 (IL-1), tumor necrosis factor-α (TNF-α), heparin-binding epidermal growth factor (HB-EGF), transforming growth factor-β (TGF-β), and fibroblast growth factors (FGF) that promote infiltration and proliferation of vascular smooth muscle cells from the media to the arterial intima. Vascular smooth muscle cells that are migrated into the intima layer results in the thickening of the arterial walls and where they transform the fatty streak into a stable plaque by secreting extracellular matrix proteins. In the advanced atherosclerotic stage, macrophages induce the release of the inflammatory cytokines and proteolytic enzymes, which results in decreased extracellular matrix production, and enhanced apoptosis within the necrotic core. Dying macrophages will then release their lipid contents and tissue factors and finally form a pro-thrombotic necrotic core which contributes to unstable plaques and their rupture is followed by intravascular blood clot formation which results in myocardial infarction and stroke [2,3,9,10].

Foam cell formation involves the disruption of normal macrophage cholesterol metabolism, a process which is regulated by a homeostatic mechanism that controls the uptake, intracellular metabolism, and efflux of cholesterol. There are studies reporting that the induction of antioxidants proteins, inhibition of receptor-induced modified low density lipoprotein (LDL) uptake and upregulation of cholesterol efflux transporters have anti-atherogenic effects. Nuclear factor erythroid 2-related factor 2 (Nrf2) is a transcription factor that is closely associated with atherosclerosis development, where it acts as a master redox switch in activating cellular antioxidant defense mechanism. Interestingly, studies reported that Nrf2 has a dual role in atherosclerosis [11,12,13]. Hence, further investigations on the role of Nrf2 in foam cells formation and atherosclerosis are required before it can be targeted for the prevention and treatment of atherosclerosis. This review will focus on the role of Nrf2 regulation in the activation of antioxidant genes, scavenger receptors, and ATP-binding cassette (ABC) transporters and their relationship in macrophage foam cells formation.

## 2. Structural Features of Nuclear Factor Erythroid 2-Related Factor 2 (Nrf2) and Regulation of Nrf2-Keap1/Antioxidant Response Elements (ARE) Signaling

**Figure 2:**
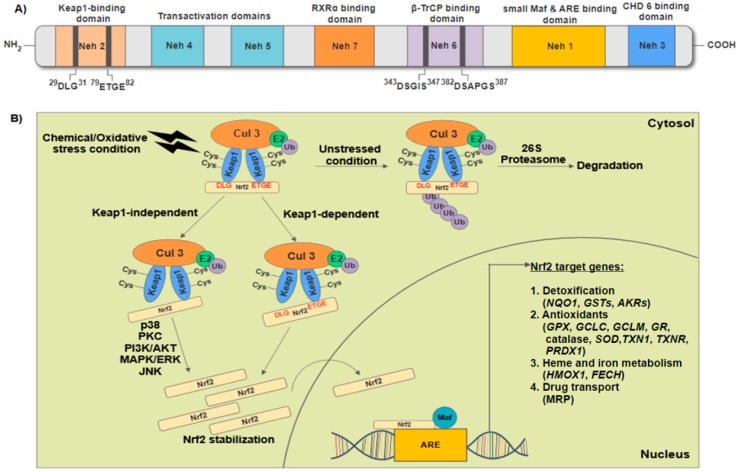
Nuclear factor erythroid 2-related factor 2 (Nrf2) regulatory pathway. (**A**) Structural Nrf2-ECH homology (Neh) 1–7 domains of human Nrf2 protein; (**B**) Keap1-dependent and Keap1-independent mediated Nrf2 regulatory pathway. Under basal condition, Nrf2 undergoes Keap-1 mediated polyubiquitination and degradation in the proteasomes. Exposure of cells to oxidative stress triggers a conformational change in Keap1 through modification of its cysteine residues, which results in the release of Nrf2 from Keap1. Apart from Keap1-dependent pathway, Nrf2 activation is also mediated by p38, PKC, PI3K/AKT, MAPK/ERK and JNK via phosphorylation of the serine or threonine residues of Nrf2. Stabilized cytosolic Nrf2 are translocated into the nucleus whereby they form heterodimers with small Maf protein and activate cell defense genes. Keap1 indicates Kelch-like ECH-associated protein 1; RXRα, retinoid X receptor α; β-TrCP, β-transducin repeat-containing protein; Maf, musculoaponeurotic fibrosarcoma; ARE, antioxidant response element; CHD 6, chromo-ATPase/helicase DNA-binding protein 6; Ub, ubiquitin; PKC; protein kinase C; PI3K/AKT, phosphatidylinositol-3-kinase; MAPK, mitogen-activated protein kinase cascades; ERK, extracellular-signal-regulated kinase; JNK, c-Jun N-terminal kinase; *NQO1*, NADPH quinine oxidoreductase 1; *GSTs*, glutathione *S*-transferases; *AKRs*, aldo-keto reductases; *GPX*, glutathione peroxidase; *GCLC*, glutamate-cysteine ligase; *GCLM*, glutamate-cysteine ligase modifier subunit; *GR*, glutathione reductase; *SOD*, superoxide dismutase; *TXN1*, thioredoxin; *TXNR*, thioredoxin reductase 1, *PRDX1*, peroxiredoxin 1; *HMOX1*, heme oxygenase (decycling) 1; *FECH*, ferrochelatase, and; MRP, multidrug resistance-associated proteins.

Nrf2 belongs to the cap “n” collar family of basic region-leucine zipper (CNC-bZIP) transcription factors that modulate the cellular redox status [14]. It regulates genes which contain antioxidant/electrophile response elements (ARE/EpRE), including antioxidant and phase II detoxification enzymes, ABC transporters and other stress response protein expression. Human Nrf2 protein consists of 605 amino acids and it contains seven unique domains (Figure 2A), also known as the Nrf2-ECH homology (Neh) domain. Each individual domain has specific function. The Neh 1 domain comprises the conserved CNC-bZIP region which is responsible for the dimerization with small musculoaponeurotic fibrosarcoma (Maf) proteins and acts as the binding site for ARE sequences. The Neh 2 domain negatively controls the activity of Nrf2 as it contains two highly conserved peptide sequences to which Kelch-like ECH-associated protein 1 (Keap1) binds; these are the high-affinity ETGE motif and the lower-affinity DLG motif. The Neh 3 domain is involved in transcriptional activation of Nrf2 by recruiting chromo-ATPase/helicase DNA-binding protein (CHD) 6. The Neh 4 and 5 domains represent the transactivation domains that interact with cAMP response element-binding protein (CREB)-binding protein (CBP) and receptor-associated coactivator (RAC) 3. The Neh 6 domain negatively controls the activity of Nrf2 because it contains two highly conserved peptide sequences to which β-transducin repeat-containing protein (β-TrCP) binds. The Neh 7 is a region that mediates the suppression of Nrf2 by preventing recruitment of coactivators to the Neh4 and Neh5 domains through protein-protein interaction between Nrf2 and the DNA-binding domain of retinoid X receptor α (RXRα) [15,16,17].

Under normal homeostatic and stress-free conditions, Nrf2 protective response is not needed. Therefore, inhibitor Keap1, an adaptor protein of a cullin3 (Cul3)-ring-box 1 (Rbx1) containing E3 ubiquitin ligase complex which targets Nrf2 for constant proteasomal degradation, maintains the cytosolic Nrf2 protein at low levels and prevent transcription of downstream target genes [18,19]. Under normal conditions, Nrf2 has a short half-life of approximately 20 min [20]. Exposure of cells to ROS, xenobiotics, heavy metals, oxLDL, electrophiles and pro-inflammatory cytokines, omega-3 polyunsaturated fatty acids (ω-3PUFA) such as docosahexaenoic acid (DHA) and eicosapentaenoic acid (EPA) [21,22,23] and natural dietary components with antioxidant properties including curcumin (turmeric), resveratrol and pterostilbene (grapes, blueberries), garlic (allicin), sulforaphane (broccoli, cruciferous) and green tea extract [22,24] results in the conformational change in Keap1 through modification of its cysteine residues. These modifications disrupt the low-affinity interaction between the Keap1 Kelch domain and Nrf2 DLG-motif, which results in stabilization of Nrf2. Consequently, these cytosolic free Nrf2 are then translocated into the nucleus whereby they form heterodimers with small Maf protein and followed by transcriptional activation of cell defense genes, resulting in increased resistance to stress, and eventually the cells oxidative status return to the basal state [24,25]. Apart from Keap-1 dependent regulation, Nrf2 activation is also mediated by protein kinases such as glycogen synthase kinase-3β (GSK-3β), phosphatidylinositol-3-kinase (PI3K)/Akt, protein kinase C (PKC), mitogen-activated protein kinase cascades (MAPK) and extracellular-signal-regulated kinase (ERK) signaling pathways via phosphorylation of the serine or threonine residues (Figure 2B) [24,26].

A growing body of evidence from both in vitro and in vivo studies has been established that the transcriptional activation of the Nrf2 signaling pathway protects the cells against oxidative/electrophilic stress which might lead to inflammation, apoptosis, premature aging, and cellular transformation. Activation of Nrf2 has shown to suppress the endothelial cell activation by inactivating p38 mitogen-activated protein (MAP) kinase activity and suppressing vascular cell adhesion molecule-1 (VCAM-1) expression which contributes to pro-inflammatory activation [27]. In vascular endothelium, atherosclerotic plaque preferentially occurs at the site of non-laminar blood flow and low fluid shear stress whereas blood flow with high fluid shears stress is shown to be atheroprotective. It is suggested that shear stress and laminar flow also suppress the endothelial cell activation and stimulates ARE expression via Nrf2 signaling pathway activation [27,28]. Several studies have provided the evidence that induction of Nrf2 by caloric restriction and resveratrol (dietary restriction mimetic) exerted the endothelial protective effect by up-regulating the expression of Nrf2 target genes [29,30]. Moreover, caloric restriction and resveratrol also induce SIRT1, a protective factor for endothelial cells that exerts anti-oxidative and anti-inflammatory effect. In ApoE null mice, endothelium-specific SIRT1 overexpression significantly showed a reduction of plaque size as compared to control [31]. Recent evidence also showed that SIRT1 interacts with Nrf2 where it significantly enhanced Nrf2 stabilization by suppressing its ubiquitination [32]. Meanwhile, caveolin-1 (Cav-1) is a negative regulator of Nrf2. Nrf2 inhibition by Cav-1 results in down-regulation of cellular antioxidant enzymes, whereas knockdown of Cav-1 leads to the dissociation of Nrf2 from Keap1, thereby enhancing the expression of antioxidant enzymes [33].

## 3. Nrf2 and Macrophage Foam Cells Formation

**Figure 3:**
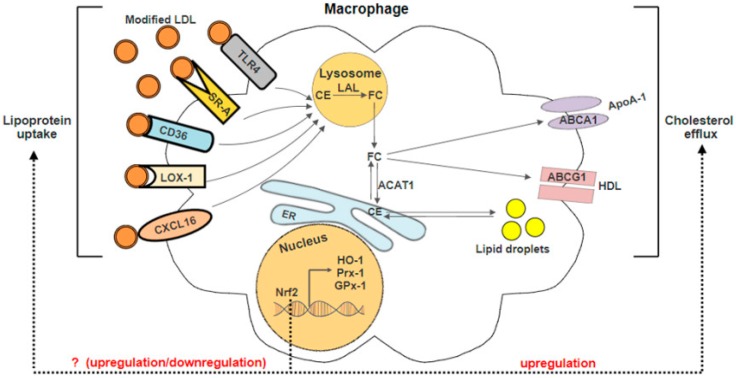
Mechanism of Nrf2 in regulating macrophage lipoprotein uptake and cholesterol efflux. Macrophages internalize modified LDL via scavenger receptors such as scavenger receptor class A (SR-A), scavenger receptor class B (CD36), lectin-type oxidized LDL receptor 1 (LOX-1), toll-like receptor 4 (TLR4) and chemokine (C-X-C motif) ligand 16 (CXCL16). The internalized modified LDL is trafficked to the lysosomes where lysosomal acid lipase (LAL) hydrolyses the excess free cholesteryl esters (CEs) to free cholesterol (FC). FC can be effluxed from the cell via ATP-binding cassette (ABC) transporters, including ABCA1 and ABCG1 or to the endoplasmic reticulum (ER). ABCA1 and ABCG1 mediate lipid efflux to lipid-free apoA-1 and HDL, respectively. In ER, the FC is re-esterified to CE by enzyme acyl-CoA: cholesterol acyltransferase (ACAT1) and stored as cytoplasmic lipid droplets. The accumulation of lipid droplets triggers the foam cells formation, which results in activation of Nrf2 and its-regulated antioxidant proteins including heme oxygenase 1 (HO-1), peroxiredoxins (Prxs), and glutathione peroxidase 1 (GPx1). Under atherogenic conditions, Nrf2 activation may up-regulate or down-regulate the expression of the lipoprotein uptake receptors. In reverse cholesterol transport, activation of Nrf2 promotes the cholesterol efflux by up-regulating the ABC transporter proteins expression. ApoA-1 indicates apolipoprotein A-1 and HDL, high-density lipoprotein.

The disruption of macrophage cholesterol metabolism, including mechanisms that control the entry, metabolism and efflux of cholesterol, will contribute to foam cells formation (Figure 3). After the internalization of modified LDLs, they are trafficked to the lysosomes where lysosomal acid lipase (LAL) hydrolyses the excess free cholesteryl esters (CEs) to free cholesterol (FC). To prevent FC mediated cell toxicity, FC is effluxed by ABC transporters or re-esterified to CE by enzyme acyl-CoA: cholesterol acyltransferase (ACAT1). Excessive CE in the endoplasmic reticulum (ER) is stored as cytoplasmic lipid droplets which subsequently trigger the formation of foam cells [9]. Studies reported that Nrf2 exhibits atheroprotective effect against oxLDL-induced foam cell formation in macrophages. Macrophages derived from Nrf2^−/−^ mice were sensitive to ROS-induced cell injury due to low antioxidants and phases II enzymes expression [34]. Low-density lipoprotein receptor-deficient (Ldlr^−/−^) mice transplanted with Nrf2^−/−^ bone marrow cells had increased macrophages migration, aoptosis, inflammation, and significant increase in atherosclerotic lesion area as compared to mice transplanted with wild-type bone marrow cells [35]. Surprisingly, some studies reported that Nrf2-deficient mice when crossed with ApoE-null hypercholesterolemic mice were protected against atherosclerosis [11,36,37]. Freigang et al. reported that Nrf2-deficient ApoE mice were protected against diet-induced atherogenesis by reducing the production of pro-inflammatory cytokine IL-1, which is responsible for the enhancement of vascular inflammation [11]. Moreover, transplantation of Nrf2-deficient bone marrow cells in ApoE knockout mouse model attenuated atherosclerotic plaque formation [38]. These evidences indicate that the activation of Nrf2 may also play a pro-atherogenic role. In the following paragraph, the role of Nrf2 in the regulation of antioxidant genes, scavenger receptors and cholesterol efflux will be discussed and summarised as schematically outlined in Figure 3 and Table 1.

### 3.1. Nrf2-Regulated Antioxidant Genes

Nrf2 exerts a plethora of protective effects against foam cell formation in the vasculature. Nrf2-regulated genes such as heme oxygenase (decycling) 1 (*HMOX1*) [39,40,41,42], peroxiredoxin (*PRDX1*) [42], glutathione peroxidase 1 (*GPX1*) [47], glutamate-cysteine ligase modifier subunit (*GCLM*) and NADPH quinine oxidoreductase 1 (*NQO1*) [48] have been implicated in the protection against atherosclerosis and oxidative stress.

Heme oxygenase 1 (HO-1) is a stress responsive protein which is highly expressed in human aortic endothelial cells [49], macrophages and smooth muscle cells [50]. The anti-atherogenic role of HO-1 has been reported in various studies. Pharmacological inhibition or knockdown of HO-1 show significant increases in lesion formation and further accelerates atherosclerosis in ApoE null mice [51], Watanabe heritable hyperlipidemic rabbits (WHHL) [52] and aortitis in chow-fed old C57BL/6 mice [53]. Apart from that, it has been demonstrated that upregulation of HO-1 genes expression by intraventricular administration of AdProT in mice has the capability to attenuate the development of atherosclerosis by reducing macrophages infiltration [54]. Likewise, the overexpression of HO-1 in rat vascular smooth muscle cells could protect the cells against oxidative stress response and the protective effect was diminished when HO-1 was inhibited by ZnPP-IX [55] HO-1 has also been reported to suppress the atherosclerotic lesion formation in ApoE mice by reducing the monocyte chemotaxis in response to LDL oxidation [49]. Interestingly, the plaque destabilizing role of HO-1 in atherosclerosis development has been explored. HO-1 induction by cobalt protoporphyrin IX (CoPPIX) suppressed the vulnerable atherosclerotic plaque development in ApoE null mice by increasing the relative cap thickness and intimal vascular smooth muscle cells while reducing the necrotic core size, as well as intimal lipids accumulation [56].

Peroxiredoxins (Prxs), a family of peroxidase enzymes, are also regulated by Nf2. Among the six isoforms of Prxs, Prx1 and Prx2 are highly expressed in various cell types including macrophages, endothelial and immune cells when exposed to oxidative stress and showed to play a major anti-atherogenic role in animal models [57,58]. It has been shown that deletion of Prx1 in mouse model significantly accelerates atherosclerosis by enhancing the leukocytes attachment and promoting the secretion of P-selectin and von Willebrand factor as well as increasing macrophage infiltration [59,60]. In addition, Prx1 has been shown to regulate lipophagic flux and macrophage cholesterol homeostasis against oxidative stress [61]. Similarly, it was shown that deficiency of Prx2 enhanced inflammatory events such as activation of p65, c-Jun, c-Jun N-terminal kinases (JNKs), and p38 MAPK [58]. Besides, Prx2-deficient mice showed higher expression of VCAM-1, intercellular adhesion molecule-1 (ICAM-1) and monocyte chemoattractant protein-1 (MCP-1), thereby increased adhesion and infiltration immune cell into the aortic intima [58].

Glutathione peroxidase-1 (GPx1), on the other hand, is a crucial antioxidant enzyme present in mammalian cells. It protects the cells against oxidative stress through detoxification of hydrogen peroxide and lipid hydroperoxides [62]. Several clinical evidences suggest a potential anti-atherogenic role for GPx1 in atherosclerosis. A prospective cohort study showed that patients with multi-vascular atherosclerosis had low GPx-1 activity and experienced a higher percentage of cardiovascular events [63]. GPx1 deficiency accelerates and modifies atherosclerotic lesion progression in ApoE-null mice [64] and diabetic ApoE-null mice [65]. In addition, disruption of GPx1 function also induces endothelial dysfunction and structural abnormalities in the myocardial vasculature [66]. Similarly, pharmacological induction of this enzyme has been shown to provide cellular protection against oxidative damage in endothelial cells [67]. These data illustrate the importance role of these enzymes in oxidative stress and atherosclerosis defense.

### 3.2. Nrf2 in Lipid Uptake

Scavenger receptors (SRs) such as scavenger receptor class A (SR-A) and scavenger receptor class B (CD36/SR-B2) are the principal receptors responsible for cholesterol uptake accounting for 75–90% of oxidatively-modified lipoproteins internalization by macrophages [68]. Moreover, lectin-type oxidized LDL receptor 1 (LOX-1) is also responsible for part of the oxLDL uptake [69]. CD36 is an 88-kDa transmembrane glycoprotein receptor. It is expressed heavily on platelets, monocytes or macrophages, adipocytes and endothelial cell [70]. Evidences suggest that the pathogenic role of oxLDL in atherosclerosis largely depends on CD36. Studies have demonstrated that genetic deletion of CD36 in ApoE double-null mice fed with standard rodent chow and Western diet were protected against atherosclerotic lesion development by decreasing the binding and uptake of modified LDL when compared to wild type [71]. Treatment with EP 80317, a competitive CD36 ligand and flavonols such as fisetin, morin, and myricetin, significantly reduced oxLDL internalisation and promoted the cholesterol efflux by inhibiting CD36 cell surface protein expression [72,73].

Apart from CD36, SR-Aalso plays a role in the uptake of modified lipid. SR-A is a 77-kDa trimeric transmembrane glycoprotein consisting of six distinct domains and it is expressed primarily on macrophages and endothelial cells lining the liver and adrenal sinusoids [74]. It was shown that circulating monocytes express a low amount of SR-A mRNA while monocytes-derived macrophages in atherosclerosis lesion have increased SR-A expression [75,76]. The absence of CD36 in hyperlipidemic mice resulted in inhibition of foam cell formation and diminished the CD36-dependent activation of JNK [77]. Silencing either SR-A or CD36 in LDL receptor-deficient apolipoprotein B100 mice exhibit had an atheroprotective effect. However, there was no beneficial effect when both receptors are silenced suggesting that compensatory activation of both receptors is sufficient for the uptake of modified LDL [78]. Similarly, ApoE-null mice lacking CD36 and SR-A were not protected against atherosclerosis development but observed a reduction in the expression of pro-inflammatory cytokines and macrophage apoptosis [79,80].

LOX-1 is a 50 kDa type II membrane glycoprotein belonging to the C-type lectin family. The receptor is highly expressed in endothelial cells, macrophages, and smooth muscle cells when the cells are exposed to oxidative stress, pro-inflammatory signals, oxLDL and others stimuli [81,82]. The pathogenic role of LOX-1 in atherosclerosis has been extensively elucidated. Silencing of LOX-1 by ginkgolide B in oxLDL stimulated endothelial cells led to inhibition ICAM-1 expression and Akt phosphorylation which results in a reduction of oxidative stress injury in endothelial cells [83]. In addition, knockdown of LOX-1 in mice fed with high cholesterol diet significantly reduced atherogenesis by sustaining endothelial function and inhibiting pro-inflammatory and pro-oxidants signals. In contrast, upregulation of LOX-1 by glucose [84] and palmitic acid [85] promotes atherosclerosis through activation of p38 MAPK and NF-κB pathways, resulting in increased VCAM-1 expression and enhancing the uptake of oxLDL by macrophages, leading to foam cell formation [86].

The role of Nrf2 in the transcriptional regulation of these scavenger receptors in macrophage has been widely studied. Absence of Nrf2 in macrophages results in upregulation of SR-A and LOX-1, and it has been implicated in the enhanced uptake of oxLDL and foam cell formation [13]. This study suggests the importance of Nrf2 in protecting against foam cells formation via regulation of receptors responsible for lipoprotein internalization in macrophages. Surprisingly, Nrf2 has been reported to exhibit pro-atherogenic properties. Several independent studies showed that deficiency of Nrf2 in ApoE-null mice developed smaller atherosclerotic plaques by reducing the uptake of acetylated-low density lipoprotein (acLDL) and reduction in the expression of the CD36 as compared to ApoE-null controls [36]. Besides, CD36 expression was increased in Nrf2^+/+^ but not in Nrf2^−/−^ macrophages [42]. Similar reports showed that Nrf2 knockout mice exhibited a reduction in atherosclerotic lesion size with decreased CD36 mRNA expression levels when compared to Nrf2 heterozygous (HET) or wild-type mice [37]. Hence, the involvement of Nrf2 pathway in the transcriptional regulation of scavenger receptors remains to be investigated.

### 3.3. Nrf2 in Cholesterol Efflux

Active processes involving ABCA1 and ABCG1 efflux transporters, as well as passive processes including simple and facilitated diffusion, are involved in macrophage reverse cholesterol transport [9,87]. In cases where mouse peritoneal macrophages were loaded with high cholesterol, ABCA1 and ABCG1 expression was enhanced while combined deficiency of both receptors resulted in foam cells accumulation and atherosclerosis [88]. Nrf2 is also involved in the regulation of ABCA1 and ABCG1 expression which will be further discussed in this section [43,44,45].

ABCA1 is a 2261-amino acid integral membrane protein that belongs to ABC subfamily A member 1. Studies have established the role of ABCA1 in the prevention of atherosclerosis in mediating lipid efflux to apolipoproteinA-1 (apoA-1), the major lipoprotein in high density lipoprotein (HDL) transport. The massive cholesterol accumulation was observed in the deletion of ABCA1 in peritoneal macrophages [89]. Suppression of ABCA1 protein degradation by small molecule inhibitor IMM-H007 promoted cholesterol efflux capacity, increased reverse cholesterol transport from macrophages to plasma and reduced the atherosclerotic plaque formation in ApoE-null mice [90]. Similarly, overexpression of human ABCA1 in LDLr^−/−^ mice exhibited an increase in cholesterol efflux to apoA-1 and inhibited the progression of the atherosclerotic lesion [91]. Another key transporter, ABCG1, which belongs to the G branch of the ABC transporter superfamily mediates cholesterol efflux to lipidated HDL particles. Deficiency of ABCG1 resulted in significant increase in the atherosclerotic lesion area in apoE^+/+^ [92] and LDLr^−/−^ mice in the early stage of atherosclerosis [93]. Likewise, upregulation of ABCA1, ABCG1 and SR-B1 in THP-1 macrophage-derived foam cells significantly reduced the cellular cholesterol content while increasing cholesterol efflux [94]. Although these findings suggested that upregulation of ABC transporters activity resulted in significant protection against atherosclerosis, some contradictory results were reported. Overexpression of ABCA1 in the liver of LDLr^−/−^ mice results in accumulation of lipoproteins, increased hepatic cholesterol concentrations, leading to enhance atherosclerosis [95]. In more advanced stages of atherosclerosis, deficiency of ABCG1 in LDLr^−/−^ mice results in delayed lesion development [93]. It is suggested that impaired cholesterol efflux from ABCG1-deficient lipid-laden macrophages, led to accelerated apoptosis and compensatory upregulation of ABCA1 expression and ApoE secretion delayed lesion progression [96,97]. Furthermore, two independent studies have shown a potential synergistic relationship between ABCA1 and ABCG1 in regulating cellular cholesterol homeostasis. Double-knockout (ABCA1^−/−^/ABCG1^−/−^)mice displayed accelerated atherosclerosis development and observed massive foam cell formation in the myocardium, lung, liver, Peyer’s patches, lymph nodes, and spleen, and increased secretion of inflammatory cytokines and chemokines [88,98,99].

It is well established that the expression of ABCA1 and ABCG1 is induced by excessive cholesterol accumulation within the cells and activation of liver X receptor and retinoid X receptor [100,101,102]. Nrf2 was recently reported to be involved in the activation of ABCA1 and ABCG1 in macrophages. Epigallocatechin-3-gallate (EGCG) has been shown to prevent TNF-α-induced nuclear factor-κB (NF-κB) activity and up-regulate ABCA1 via Nrf2/Keap1 pathway in macrophage foam cells [45]. Similarly, *Tert*-butylhydroquinone (tBHQ) was found to reduce calpain, a protease that promotes ABCA1 proteolysis via activation of the Nrf2/HO-1 pathway [44]. Treatment with Tanshinone IIA ameliorated lipid accumulation in macrophage foam cells by reducing the expression of SR-A and increasing the expression of ABCA1 and ABCG1 via activation of ERK/Nrf2/HO-1 pathway [43]. These data suggest that new therapeutic strategies aimed at increasing cholesterol efflux by enhancing macrophage ABCA1 and ABCG1 expression are likely to be beneficial for the treatment of atherosclerosis.

## 4. Recent Insights of Nrf2 in Macrophage Foam Cells Formation

In the past few years, researches have demonstrated the anti-atherogenic role of Nrf2 in foam cells formation. Recently, Cui et al. demonstrated that treatment of atherosclerotic Wistar rats with Urolithin A attenuated foam cell formation by inducing upregulation of SR-B1-mediated cholesterol reverse transport via the Nrf2 pathway [103]. Jongstra-Bilen et al. reported that Nrf2 plays a critical role in reducing the expression of a subset of pro-inflammatory genes including IL-1β, IL-6, and CCL5 in oxLDL-loaded cells [104]. Moreover, Xie et al. reported that H_2_S reduced foam cell formation by inhibiting superoxide, VCAM-1, and ICAM-1 generation and enhanced HO-1 expression via Nrf2 activation in wild type mice but not in Nrf2^−/−^ mice [105]. This is consistent with another study showing that lipoicmethylenedioxyphenol (LMDP) inhibited macrophage chemotaxis via Nrf2 activation and subsequent reduction of the atherosclerotic lesion [106]. These findings strongly support that reduction of atherosclerotic lesion by H_2_S and LMDP treatments is mediated by activating the Nrf2 signaling pathway. Apart from drug administration, sonodynamic therapy (SDT) exhibited a protective role against atherosclerosis by inducing HO-1 expression in macrophages through activation of Nrf2 signaling pathway [107]. Hence, these evidences suggested that Nrf2 pathway could be a valuable therapeutic target for the treatment and prevention of atherosclerosis.

## 5. Conclusions

Nrf2 is an essential transcription factor involved in cellular antioxidant defense mechanism. It plays significant roles in regulating the expression of target genes which are involved in cholesterol influx and release including antioxidant enzymes, scavenger receptors, and ABC transporter proteins. These Nrf2-regulated genes expression are responsible for macrophages cholesterol loading and subsequently foam cells formation. The anti-atherogenic function of Nrf2 has been demonstrated in both in vitro and in vivo studies. Nevertheless, reports on the paradoxical role of Nrf2 warrants additional investigations to be carried out in order to verify the significance of Nrf2 pathway associated with foam cells/fatty streak development before it is developed as a potentially novel therapeutic target.

## Figures and Tables

**Table 1:** The pro and anti-atherogenic role of Nrf2 in regulating target genes involved in macrophage foam cells formation.

Targets	Experimental Model/Cell Line	Study Finding	Properties	Source
Antioxidant genes	HASMC	Nrf2^−/−^, ↓HO-1 & Prx-1	Anti-atherogenic	[39]
	RAW264.7 & Nrf2^−/−^ mice	Nrf2^−/−^, ↓HO-1, ↑IL-1β & IL-6	Anti-atherogenic	[40]
	HAECs, HMECs, Human mesangial cells & U937 cells	Nrf2^+/+^, ↑HO-1 & GPx, ↑intracellular GSH level, ↓MCP-1 & VCAM-1, ↓adhesion activity	Anti-atherogenic	[41]
	Mouse peritoneal macrophages & SMCs	Nrf2^+/−^, ↑ stress protein A170, HO-1 & Prx-1	Anti-atherogenic	[42]
Cholesterol uptake receptors	LDLR^−/−^ mice	Nrf2^−/−^, ↑atherosclerotic lesions, ↑uptake of acetylated and malondialdehyde-modified LDLs, ↑expression of TLR4, SR-A, LOX-1 & CXCL16	Anti-atherogenic	[13]
	ApoE^−/−^ mice	Nrf2^−/−^, ↓CD36, ↓ cholesterol influx	Pro-atherogenic	[37]
	ApoE^−/−^ mice	Nrf2^−/−^, ↓atherosclerotic plaques, ↓uptake of acLDL, ↓expression of CD36	Pro-atherogenic	[36]
	Mouse peritoneal macrophages	Nrf2^+/+^, ↑CD36	Pro-atherogenic	[42]
Cholesterol efflux receptors	THP-1 cells & primary human macrophages	Tan-induced Nrf2 activation, ↑HO-1, ↓SR-A, ↑ABCA1 & ABCG1	Anti-atherogenic	[43]
	THP-1 cells	tBHQ-induced Nrf2 & HO-1 activation, ↑ABCA1, ↑cholesterol efflux	Anti-atherogenic	[44]
	THP-1 cells	EGCG-induced Nrf2 activation, ↓TNF-α-induced NF-κB activation, ↑ABCA1	Anti-atherogenic	[45]
Proinflammatory cytokines & others mediators	U937 cells	Nrf2^−/−^, ↑IL-1β, IL-6 & TNFα, ↑MCP-1, ↑ROS & ER stress markers expression	Anti-atherogenic	[46]
	LDLR^−/−^ mice	Nrf2^−/−^, ↑ MCP-1, IL-6 & TNF-α	Anti-atherogenic	[13]
	ApoE^−/−^ mice	Nrf2^−/−^, ↓atherosclerotic lesions, ↓cholesterol crystal-induced IL-1 production	Pro-atherogenic	[11]

HASMC indicates human aortic smooth muscle cells; HAECs, human aortic endothelial cells; HMECs, human dermal microvascular endothelial cells; SMCs, smooth muscle cells; tBHQ, *tert*-butylhydroquinone. Note: An upward-pointing arrow (↑) indicates increase; a downward-pointing arrow (↓) indicates decrease.
